# Sinus heart rate post pulmonary vein ablation and long-term risk of recurrences

**DOI:** 10.1007/s00392-020-01765-z

**Published:** 2020-11-12

**Authors:** Gesa von Olshausen, Ott Saluveer, Jonas Schwieler, Nikola Drca, Hamid Bastani, Jari Tapanainen, Tara Bourke, Astrid Paul-Nordin, Göran Kennebäck, Per Insulander, Mats Jensen-Urstad, Frieder Braunschweig

**Affiliations:** Department of Cardiology, Karolinska University Hospital, S1:02, 17176 Solna, Stockholm Sweden

**Keywords:** Atrial fibrillation, Pulmonary vein isolation, Parasympathetic denervation, Sinus heart rate, Recurrence

## Abstract

**Purpose:**

Cather ablation is known to influence the autonomic nervous system. This study sought to investigate the association of sinus heart rate pre-/post-ablation and recurrences in patients with atrial fibrillation undergoing pulmonary vein isolation (PVI).

**Methods:**

Between January 2012 and December 2017, data of 482 patients undergoing their first PVI were included. Sinus heart rate was recorded before (PRE), directly post-ablation (POST) and 3 months post-ablation (3 M). All patients were screened for atrial tachyarrhythmia recurrences during the one-year follow-up.

**Results:**

In the total study cohort, the mean resting sinus heart rate at PRE [mean 57.9 bpm (95% CI 57.1–58.7 bpm)] increased by over 10 bpm to POST [mean 69.4 bpm (95% CI 68.5–70.3 bpm); *p* < 0.001] followed by a slight decrease at 3 M [mean 67.3 bpm (95% CI 66.4–68.2 bpm)] but still remaining higher compared to PRE (*p* < 0.001). This pattern was observed in patients with and without recurrences at POST and 3 M (both *p* < 0.001 compared to PRE). However, at 3 M the mean sinus heart rate was significantly lower in patients with compared to patients without recurrences (*p* = 0.031). In this regard, patients with a heart rate change < 11 bpm (PRE to 3 M) or, as an alternative parameter, patients with a heart rate < 60 bpm at 3 M had a significantly higher risk of recurrences compared to the remaining patients (Hazard ratio (HR) 1.82 (95% CI 1.32–2.49), *p* < 0.001 and HR 1.64 (95% CI 1.20–2.25), *p* = 0.002, respectively).

**Conclusion:**

Our study confirms the impact of PVI on cardiac autonomic function with a significant sinus heart rate increase post-ablation. Patients with a sinus heart rate change < 11 bpm (PRE to 3 M) are at higher risk for recurrences during one-year post-PVI.

**Electronic supplementary material:**

The online version of this article (10.1007/s00392-020-01765-z) contains supplementary material, which is available to authorized users.

## Introduction

Atrial fibrillation is the most common arrhythmia of clinical significance and its prevalence is rising worldwide [1]. Catheter ablation aiming at pulmonary vein isolation (PVI) has become an effective treatment option in patients with symptomatic atrial fibrillation. However, recurrences of atrial tachyarrhythmias after initially successful catheter ablation are common and often require re-do ablations [2]. Despite increasing knowledge about the risk factors for recurrences, identifying patients at risk for recurrence that would benefit from close monitoring remains challenging. The autonomic nervous system has been shown to play a pivotal role in the pathogenesis of atrial fibrillation [3]. In this regard, it has been shown that patients with atrial fibrillation and a low resting sinus heart rate prior to ablation seem to carry a higher risk for recurrence of atrial fibrillation post-ablation [4, 5]. It has been suggested that catheter ablation has an attenuating effect on parasympathetic activity [6, 7] leading to an increased resting heart rate post-ablation. In this context, it has been shown that a low sinus heart rate post-ablation is associated with a significantly higher recurrence risk of atrial fibrillation during follow-up [8–10]. However, studies were either limited by sample size or timepoints for significant changes in sinus heart rate differed. Hence, the aim of this study was to investigate the association of sinus heart rate pre-/post-ablation and recurrences in patients undergoing PVI for atrial fibrillation in a large cohort.

## Methods

### Study population

All patients who underwent catheter ablation for atrial fibrillation at the Karolinska University Hospital between January 2012 and December 2017 were included provided that a first-time PVI procedure using the radiofrequency technique was performed without major complications such as cardiac tamponade, cerebrovascular events, major bleeding and AV fistula. Patients subjected to additional ablation lines in the right/left atrium or ablation of complex fractionated atrial electrograms were excluded. Patients with a pacemaker or implantable cardioverter defibrillator were also excluded. Complete follow-up information had to be available for 12 months post-PVI. Relevant patient characteristics and procedural details were prospectively collected at the time of the ablation procedure and recorded in a computerized database. All patient data and follow-up information were derived from the digital medical record system (TakeCare, CompuGroup Medical Sweden, Uppsala, Sweden) which covers most of the hospitals and medical practices in Stockholm county.

According to the 2016 ESC guidelines for the management of atrial fibrillation [11], atrial fibrillation was classified as paroxysmal if self-terminating (in most cases within 48 h) or if atrial fibrillation episodes were cardioverted within 7 days. Atrial fibrillation was classified as persistent if it lasted longer than 7 days, including episodes that were terminated by cardioversion (either with drugs or by direct current cardioversion) after 7 days or more.

### Catheter ablation procedure

Oral anticoagulation therapy was prescribed at least one month before the procedure. Transesophageal echocardiography prior to the procedure was performed to exclude left atrial appendage thrombus in all patients. The ablation procedures were performed under conscious sedation and analgesia. Throughout the procedure, a continuous infusion of heparin was maintained to achieve an activated clotting time (ACT) of > 300 s and ACT measurements were routinely done every 30 min. All patients underwent sole circumferential PVI as described before at our institution [12]. In brief, vascular access was obtained using the right and/or left femoral vein. Under fluoroscopic guidance trans-septal access to the left atrium was established through which the radiofrequency ablation catheter (Biosense-Webster Inc., Diamond Bar, CA, USA) and circular mapping catheter (Lasso, Biosense-Webster Inc., Diamond Bar, CA, USA) guided by a 3-dimensional mapping system (Carto, Biosense-Webster Inc., Diamond Bar, CA, USA or NavX, St. Jude Medical Inc., St. Paul, MN, USA) were advanced into the left atrium. Circumferential lesions were created to surround the right and left pulmonary veins (PV) with a 3.5 mm irrigated-tip catheter (Biosense-Webster Inc., Diamond Bar, CA, USA). RF energy was applied with a power between 25 and 35 W, with an irrigation rate of 10 to 40 mL/min.

Acute procedural success was defined as an entrance and exit block at least 20 min after initial PV isolation, documented with a circular mapping catheter. Those PVs with acute reconnection were re-isolated. Application of adenosine to assess for dormant PV conduction after ablation was not performed. All patients who underwent ablation were treated with the same approach.

### Post-ablation follow-up

Post-ablation, patients were closely monitored for post-procedural complications and discharged home after 24 h. Oral anticoagulation was continued for at least 3 months post-ablation. Further use of oral anticoagulation was determined according to the ESC guidelines [11]. At the discretion of the treating physician, prescription of anti-arrhythmic drugs (AADs) during the 3 months blanking period was performed to favor reverse electrical and structural atrial remodelling. AADs were stopped in all symptom-free patients not later than the end of the blanking period.

For heart rate assessment, resting 12-lead electrocardiograms (ECGs) were recorded within 24 h before ablation (PRE), within 24 h post-ablation (POST) and 3 months post-ablation (3 M). Patients were only included when sinus rhythm was present during these ECG measurements.

For ECG registration, patients had to lay down in supine position. After 3–5 min of rest, the ECG was recorded. ECG registration was at a paper speed of 50 mm/s (PHILIPS, Amsterdam, Netherlands) and digitally stored in the digital medical record system.

Patients were followed-up for one year with clinical visits in the outpatient clinic and at medical practices scheduled at 3-, 6- and 12-months post-procedure. An ambulatory ECG and/or 24-h Holter ECG (at least one Holter ECG during follow-up) was routinely obtained during follow-up visits as well as during unscheduled ambulatory visits related to arrhythmia recurrences. Documentation of arrhythmic episodes was based on ECG, Holter-ECG or event loop recorder (when available). Recurrences were defined as any documented atrial tachyarrhythmia (atrial fibrillation, atrial flutter, atrial tachycardia) lasting > 30 sec occurring after the 3 months blanking period.

### Statistical analysis

Continuous variables are presented as mean ± standard deviation or 95% confidence intervals and were compared using Student’s t-tests and ANOVAs. Continuous variables presented as median and interquartile range were compared using Mann–Whitney tests. Categorical variables are expressed as frequencies/percentages and were compared by Chi-square tests. The Kaplan–Meier method was used for building event curves. Hazard ratios with 95% confidence intervals and *p* values from Cox regression and Log-Rank analyses are provided. All statistical tests and confidence intervals were 2-sided, with a significance level of 0.05. Univariate and multivariable backward logistic regression analyses were performed. The multivariable model considered factors associated with a *p* value < 0.05 in univariate analysis. Log-rank optimization was performed to determine a cutoff which may have prognostic significance for recurrence. The optimal cut-off was considered as the sinus heart rate change (from PRE to 3 M) separating the population in two groups of which the log-rank comparison revealed the highest chi-square statistic. This was performed using the R package ‘maxstat’ Version 0.7–25 in R (Version 3.6.1) [13]. All other statistical analyses were performed using SPSS software, version 25 (IBM Corp., Armonk, New York).

## Results

**Fig. 1:**
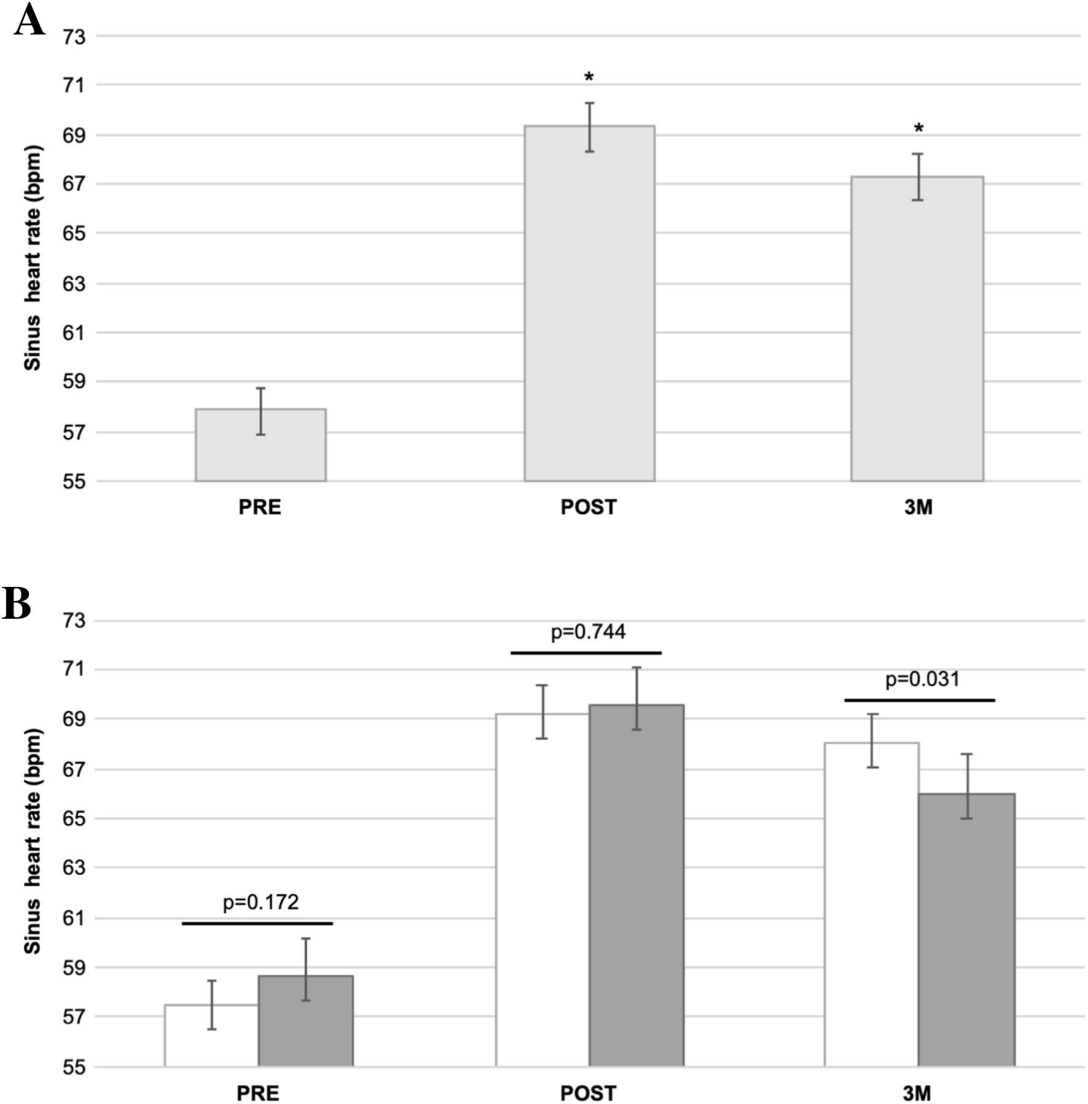
**a** Mean sinus heart rate (with 95% confidence interval) of the total study population at PRE, POST and 3 M. **b** Mean sinus heart rate (with 95% confidence interval) at PRE, POST and 3 M in patients without (white bars) and with (grey bars) recurrence. * *p* < 0.001 as compared to PRE

Between January 2012 and December 2017, 1836 patients underwent ablation for atrial fibrillation at the Karolinska University Hospital. From this patient cohort, 482 patients fulfilled the inclusion criteria and were included in the analysis (Figure 1, supplementary). Patients were divided into two groups dependent on experiencing recurrences [173 patients (35.9%)] vs. no recurrences [309 patients (64.1%)]. Patients with recurrences compared to patients without recurrences had a significantly higher body mass index (27.4 vs. 26.4 kg/m2, *p* = 0.002), more persistent than paroxysmal atrial fibrillation (46.8% vs. 15.5%, *p* < 0.001), a longer history of atrial fibrillation (5.0 vs. 4.0 years, *p* < 0.001), a higher CHA_2_DS_2_-VASc Score (*p* = 0.033), more arterial hypertension (53.2% vs. 38.8%, *p* = 0.003), more diabetes mellitus (9.2% vs. 4.2%, *p* = 0.029), a larger left atrial size in the parasternal long axis (39.9 vs. 38.9 mm, *p* = 0.012), received more beta-blocker at PRE (69.4% vs. 60.2%, *p* = 0.049) as well as at 3 M (71.1% vs. 57.9%, *p* = 0.004) and received more other AADs at 3 M (*p* = 0.047). All baseline and procedural characteristics of patients with and without recurrences are presented in Table 1.

**Table 1:** Baseline and procedural characteristics of patients with and without recurrences

Baseline characteristics	All patients (*n* = 482)	No recurrence (*n* = 309)	Recurrence (*n* = 173)	*p* value
Age (years)	59.3 ± 10.1	59.1 ± 10.3	59.5 ± 9.8	0.650
Male, *n* (%)	319 (66.2)	214 (69.3)	105 (60.7)	0.071
BMI (kg/m2)	26.8 ± 3.5	26.4 ± 3.4	27.4 ± 3.6	0.002
Type of atrial fibrillation				< 0.001
Paroxysmal, *n* (%)	353 (73.2)	261 (84.5)	92 (53.2)	
Persistent, *n* (%)	129 (26.8)	48 (15.5)	81 (46.8)	
Duration of atrial fibrillation in the past, years	4.2 (2.0; 7.3)	4.0 (2.1; 6.0)	5.0 (2.9; 10.0)	< 0.001
Number of failed AADs	1.1 ± 0.7	0.9 ± 0.6	1.3 ± 0.8	< 0.001
CHA_2_DS_2_-VASc Score, *n* (%)				0.033
0	133 (27.6)	89 (28.8)	44 (25.4)	
1	158 (32.8)	112 (36.2)	46 (26.6)	
2	108 (22.4)	59 (19.1)	49 (28.3)	
> = 3	83 (17.2)	49 (15.9)	34 (19.7)	
Congestive heart failure, *n* (%)	32 (6.6)	19 (6.1)	13 (7.5)	0.571
Structural heart disease				0.792
Ischemic cardiomyopathy, *n* (%)	15 (3.1)	11 (3.6)	4 (2.3)	
Dilated cardiomyopathy, *n* (%)	8 (1.7)	6 (1.9)	2 (1.2)	
Hypertrophic cardiomyopathy, *n* (%)	6 (1.2)	4 (1.3)	2 (1.2)	
Arterial hypertension, *n* (%)	212 (44.0)	120 (38.8)	92 (53.2)	0.003
Diabetes mellitus, *n* (%)	29 (6.0)	13 (4.2)	16 (9.2)	0.029
Hyperlipidemia, *n* (%)	80 (16.6)	50 (16.2)	30 (17.3)	0.799
Left atrial size, parasternal long axis (mm)	39.3 ± 4.3	38.9 ± 4.1	39.9 ± 4.6	0.012
Left ventricular ejection fraction (%)	58.1 ± 4.5	58.4 ± 4.4	57.7 ± 4.6	0.108
Time of RF energy delivery (sec)	2677.0 ± 946.8	2642.5 ± 891.6	2736.1 ± 1038.3	0.354
Procedure time (min)	172.2 ± 50.5	167.4 ± 47.5	179.5 ± 54.7	0.009
Fluoroscopy time (min)	15.6 ± 9.6	15.1 ± 9.5	16.4 ± 9.5	0.096
Beta-blocker at PRE, *n* (%)	306 (63.5)	186 (60.2)	120 (69.4)	0.049
Other AADs at PRE				0.871
Dronedarone, *n* (%)	60 (12.4)	37 (12.0)	23 (13.3)	
Flecainid, *n* (%)	56 (11.6)	33 (10.7)	23 (13.3)	
Sotalol, *n* (%)	43 (8.9)	29 (9.4)	14 (8.1)	
Amiodarone, *n* (%)	24 (5.0)	16 (5.2)	8 (4.6)	
Disopyramid, *n* (%)	8 (1.7)	4 (1.3)	4 (2.3)	
Propafenon, *n* (%)	1 (0.2)	1 (0.3)	0 (0.0)	
Beta-blocker at 3 M, *n* (%)	302 (62.7)	179 (57.9)	123 (71.1)	0.004
Other AADs at 3 M				0.047
Dronedarone, *n* (%)	27 (5.6)	13 (4.2)	14 (18.1)	
Flecainid, *n* (%)	19 (3.9)	7 (2.3)	12 (6.9)	
Sotalol, *n* (%)	21 (4.4)	12 (3.9)	9 (5.2)	
Amiodarone, *n* (%)	13 (2.7)	8 (2.6)	5 (2.9)	
Disopyramid, *n* (%)	2 (0.4)	1 (0.3)	1 (0.6)	
Propafenon, *n* (%)	0 (0.0)	0 (0.0)	0 (0.0)	

*BMI* body mass index, *AAD* antiarrhythmic drug, *RF* radiofrequency

^a^Non-normally distributed continuous variables are expressed as the median and interquartile range (25th and 75th percentile)

**Fig. 2:**
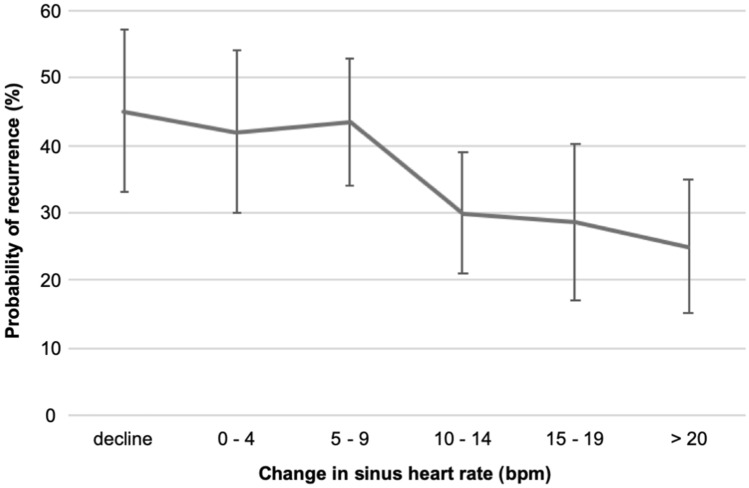
Sinus heart rate change (PRE to 3 M) with a corresponding probability of recurrence (with 95% confidence interval)

**Fig. 3:**
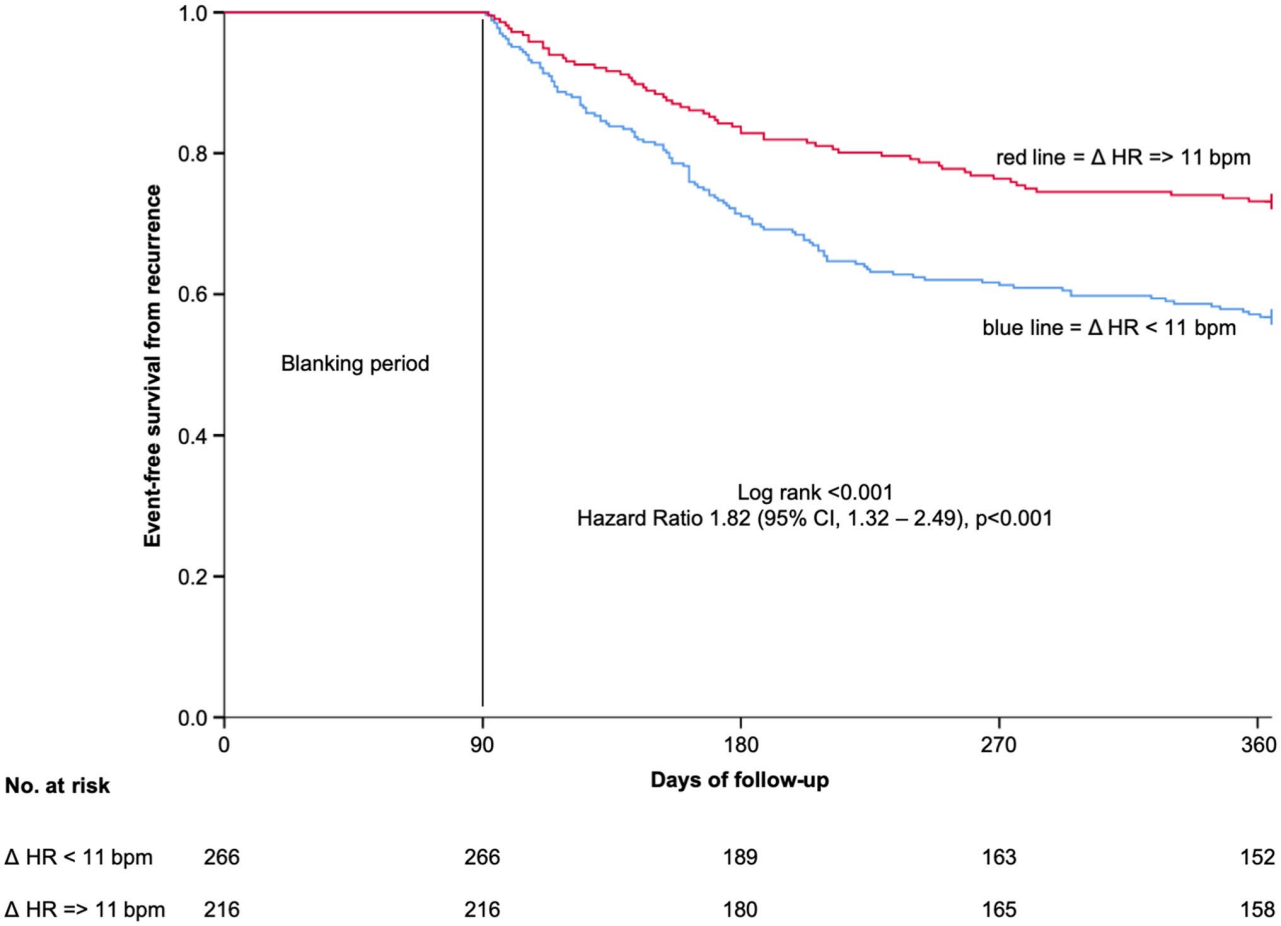
Kaplan–Meier analysis of event-free survival from recurrence in patients with a sinus heart rate change < 11 bpm compared to >= 11 bpm (PRE to 3 M) after a one-year follow-up

The mean resting sinus heart rate of the total study population was 57.9 beats per minute (bpm) before ablation (PRE) (95% CI 57.1–58.7 bpm). Directly post-ablation (POST) it significantly increased by 11.5 bpm (mean 69.4 bpm (95% CI 68.5–70.3 bpm); *p* < 0.001) (Fig. 1a) with a distribution of heart rate change (PRE to POST) as provided in Fig. 2, supplementary. After 3 months post-ablation (3 M), the heart rate slightly decreased but remained markedly higher as compared to PRE (mean 67.3 bpm (95% CI 66.4–68.2 bpm); *p* < 0.001) (Fig. 1a) with a distribution of heart rate change (PRE to 3 M) as provided in Figure 2, supplementary. This increasing pattern was observed in patients with as well as without recurrences at POST and 3 M respectively (both *p* < 0.001 as compared to PRE). However, in patients with recurrences the mean heart rate was significantly lower compared to patients with no recurrences at 3 M (mean 66.0 bpm (64.4–67.6 bpm) vs. mean 68.1 bpm (95% CI 67.0–69.2 bpm); *p* = 0.031) (Fig. 1b). Due to this difference at 3 M, we analyzed the heart rate change (PRE to 3 M) and the corresponding probability of recurrence. In this analysis, an increase of sinus heart rate change went along with a decreased probability of recurrence (Hazard ratio (HR) 0.85 (95% CI 0.78–0.94); *p* = 0.001) (Fig. 2). In addition, we performed a Log-Rank optimization to determine a cut-off of heart rate change (PRE to 3 M) which maximized the discrimination for recurrences. This analysis revealed a cut-off of 11 bpm. According to this cut-off, 266 patients (55.2%) had a heart rate change < 11 bpm and 216 patients (44.8%) >= 11 bpm. Treatments with beta-blocker and other antiarrhythmic drugs (AADs) as well as procedural characteristics were similar in both groups (Table 2). Recurrences occurred in 115 patients (43.2%) in the heart rate change < 11 bpm group compared to 58 patients (26.9%) in the heart rate change >= 11 bpm group (HR 1.82 (95% CI 1.32–2.49), *p* < 0.001) during a one-year follow-up. The corresponding Kaplan–Meier-curve is provided in Fig. 3. In multivariable analysis, a heart rate change < 11 bpm (PRE to 3 M) remained independently associated with recurrences (Table 1, supplementary).

**Table 2:** Treatment with beta-blocker and other antiarrhythmic drugs as well as procedural characteristics in patients with a sinus heart rate change < 11 bpm and >= 11 bpm (PRE to 3 M)

Variable	Sinus heart rate change < 11 bpm (PRE to 3 M) (*n* = 266)	Sinus heart rate change > = 11 bpm (PRE to 3 M) (*n* = 216)	*p* value
Beta-blocker at PRE, *n* (%)	170 (63.9)	136 (63.0)	0.850
Other AADs at PRE			0.408
Dronedarone, *n* (%)	38 (14.3)	22 (10.2)	
Flecainid, *n* (%)	26 (9.8)	30 (13.9)	
Sotalol, *n* (%)	23 (8.6)	20 (9.3)	
Amiodarone, *n* (%)	16 (6.0)	8 (3.7)	
Disopyramid, *n* (%)	5 (1.9)	3 (1.4)	
Propafenon, *n* (%)	0 (0.0)	1 (0.5)	
Beta-blocker at 3 M, *n* (%)	176 (66.2)	126 (58.3)	0.088
Other AADs at 3 M			0.093
Dronedarone, *n* (%)	19 (7.1)	8 (3.7)	
Flecainid, *n* (%)	11 (4.1)	8 (3.7)	
Sotalol, *n* (%)	14 (5.3)	7 (3.2)	
Amiodarone, *n* (%)	11 (4.1)	2 (0.9)	
Disopyramid, *n* (%)	1 (0.4)	1 (0.5)	
Propafenon, *n* (%)	0 (0.0)	0 (0.0)	
Time of RF energy delivery (sec)	2710.2 ± 979.1	2634.2 ± 906.1	0.446
Procedure time (min)	174.4 ± 50.8	168.4 ± 49.9	0.145
Fluoroscopy time (min)	15.3 ± 8.7	15.9 ± 10.4	0.826

*AAD* antiarrhythmic drug, *RF* radiofrequency

**Fig. 4:**
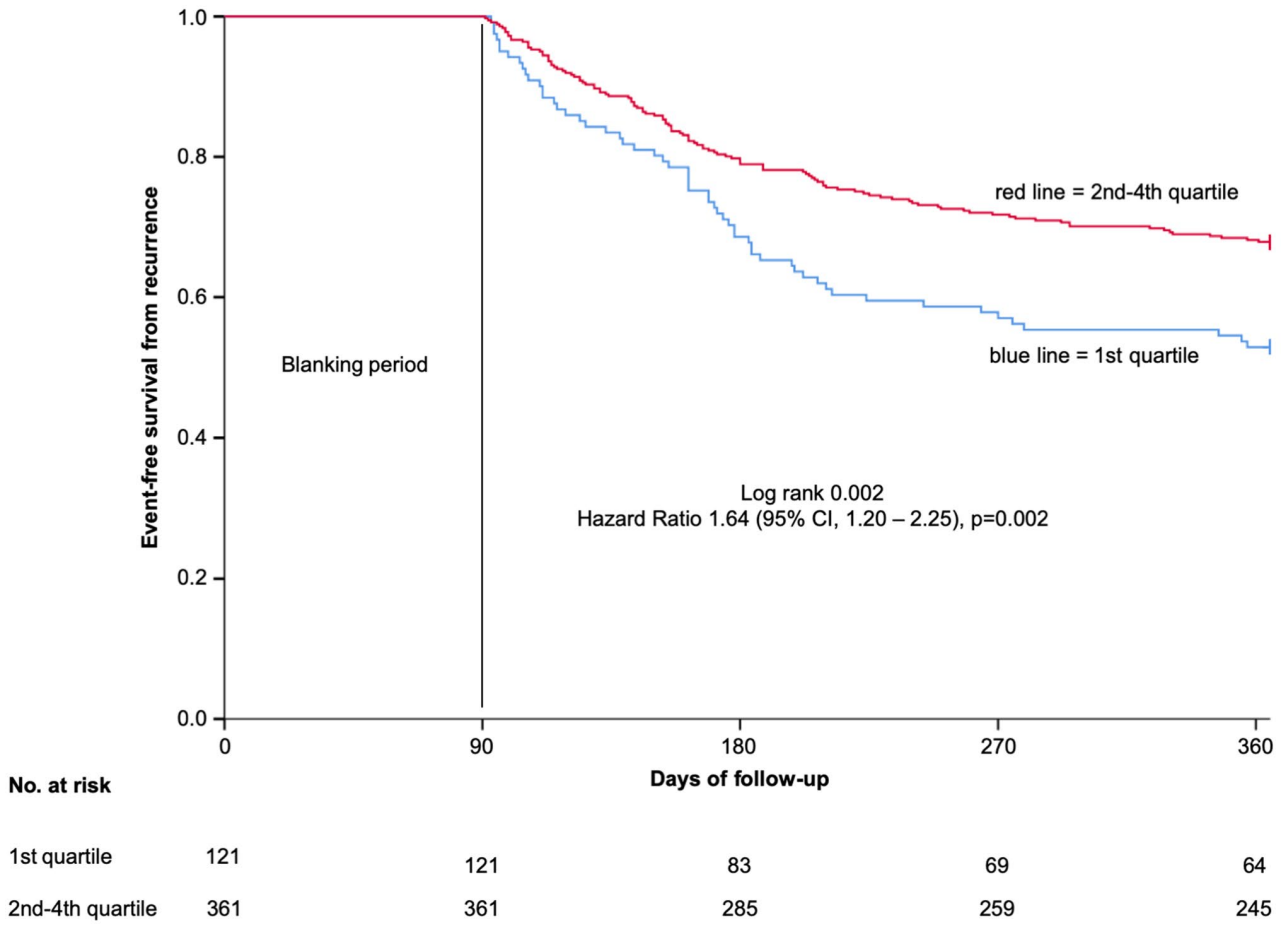
Kaplan–Meier analysis of event-free survival from recurrence in patients within the 1st quartile compared to 2nd—4th quartile at 3 M after a one-year follow-up

For further analysis, we divided all patients into quartiles regarding to their sinus heart rate at 3 M. Patients in the 1st quartile had a significantly increased risk of recurrences compared to the 4th quartile (HR 1.59, 95% CI 1.05–2.38, *p* = 0.027) while the other quartiles showed a comparable recurrence risk (2nd quartile HR 1.02, 95% CI 0.66–1.58, *p* = 0.938 and 3rd quartile HR 0.88, 95% CI 0.56–1.39, *p* = 0.588) during a one-year follow-up (Table 3; corresponding Kaplan–Meier curve Figure 3, supplementary). In a direct comparison, patients in the 1st quartile had a significantly higher risk of recurrences compared to patients in the 2nd—4th quartile (57 patients (47.1%) vs. 116 patients (32.1%); HR 1.64 (95% CI 1.20–2.25), *p* = 0.002) (Fig. 4). Treatments with beta-blocker and other AADs as well as procedural characteristics were similar in patients of the 1st quartile compared to patients in the 2nd—4th quartile (Table 4). In multivariable analysis, a heart rate < 60 bpm at 3 M remained independently associated with recurrences (Table 2, supplementary).

**Table 3:** Patients divided into quartiles regarding their sinus heart rate at 3 M and their risk of recurrence development

3 M	Hazard ratio	95% CI	*p* value
1st quartile: heart rate 43—59 bpm	1.59	1.05–2.38	0.027
2nd quartile: heart rate 60—66 bpm	1.02	0.66–1.58	0.938
3rd quartile: heart rate 67—72 bpm	0.88	0.56–1.39	0.588
4th quartile: heart rate 73—102 bpm	reference	–	–

*CI* confidence interval

**Table 4:** Treatment with beta-blocker and other antiarrhythmic drugs as well as procedural characteristics in patients of the 1st sinus heart rate quartile and 2nd—4th sinus heart rate quartile at 3 M

Variable	Sinus heart rate 1st quartile (3 M) (*n* = 121)	Sinus heart rate 2nd—4th quartile (3 M) (*n* = 361)	*p* value
Beta-blocker at PRE, *n* (%)	76 (62.8)	230 (62.7)	0.913
Other AADs at PRE			0.910
Dronedarone, *n* (%)	14 (11.6)	46 (12.7)	
Flecainid, *n* (%)	16 (13.2)	40 (11.1)	
Sotalol, *n* (%)	12 (9.9)	31 (8.6)	
Amiodarone, *n* (%)	7 (5.8)	17 (4.7)	
Disopyramid, *n* (%)	3 (2.5)	5 (1.4)	
Propafenon, *n* (%)	0 (0.0)	1 (0.3)	
Beta-blocker at 3 M, *n* (%)	81 (66.9)	221 (61.2)	0.279
Other AADs at 3 M			0.126
Dronedarone, *n* (%)	7 (5.8)	20 (5.5)	
Flecainid, *n* (%)	9 (7.4)	10 (2.8)	
Sotalol, *n* (%)	7 (5.8)	14 (3.9)	
Amiodarone, *n* (%)	5 (4.1)	8 (2.2)	
Disopyramid, *n* (%)	1 (0.8)	1 (0.3)	
Propafenon, *n* (%)	0 (0.0)	0 (0.0)	
Time of RF energy delivery (sec)	2599.3 ± 910.0	2701.3 ± 958.3	0.273
Procedure time (min)	173.8 ± 49.0	171.0 ± 51.0	0.586
Fluoroscopy time (min)	15.7 ± 8.7	15.5 ± 9.7	0.972

*AAD* antiarrhythmic drug, *RF* radiofrequency

### Consistency analysis

To investigate whether the observed findings are independent of antiarrhythmic drug effects, we performed a consistency analysis only considering patients without any treatment of beta-blocker or other antiarrhythmic drugs (*n* = 79). In this patient cohort, the mean resting sinus heart at PRE (59.2 bpm at (95% CI 56.9–61.6 bpm) increased by over 10 bpm to POST (mean 69.6 bpm (95% CI 67.3–71.9 bpm); *p* < 0.001) and remained high also at 3 M [mean 69.4 bpm (95% CI 66.9–72.0 bpm)] compared to PRE (*p* < 0.001). (Figure 4, supplementary). Of these patients, 62 patients (78.5%) remained free from recurrences and 17 patients (21.5%) developed recurrences.

We further analyzed the heart rate change and the corresponding probability of recurrence. The relation between sinus heart rate change (PRE to 3 M) and the probability of recurrence [HR 0.86 (95% CI 0.65–1.15); *p* = 0.306] as well as the risk increase according to the sinus heart rate change cut-off of 11 bpm [HR 1.80 (95% CI 0.67–4.88), *p* = 0.246 (Figure 5, supplementary) were similar compared to the primary analysis. However, they lacked statistical significance most likely due to the small sample size and number of events.

## Discussion

In this study of patients undergoing a first-time PVI procedure, we found that the mean resting heart rate significantly increased at POST and 3 M compared to PRE. However, only at 3 M, there was a significant difference in mean heart rate being lower in patients with compared to patients without recurrences. In this regard, patients with a heart rate change < 11 bpm (PRE to 3 M) had a significantly higher risk of recurrences compared to the remaining patients. As an alternative marker, a heart rate < 60 bpm at 3 M was associated with an unfavorable outcome in terms of recurrences. Both variables remained independently associated with multivariable analysis.

Our study confirms the significant impact of PVI on cardiac autonomic function with a mean heart rate increase by over 10 bpm at POST which is in line with previously published results [5, 7–9]. This effect is suggested to be induced by parasympathetic denervation owing to PVI ablation [6, 7] which is known to influence the sinus rate, the atrial refractory period and atrioventricular conduction [14]. Other contributing factors may be postoperative stress and pain as well as increased fluid load associated with the use of irrigated ablation catheters.

Interestingly, at 3 M the mean heart rate was lower among patients with recurrent atrial fibrillation compared to those without while we did not observe any difference in mean heart rate at PRE or POST. This is in contrast to published results, where this difference in mean heart rate among patients with and without recurrence was already observed directly post-ablation [8]. While the effect of parasympathetic denervation of PVI might be similar directly post-ablation provided that same ablation techniques have been applied to all patients, lesion maturation with potential restitution during the first weeks or months post-ablation might modify the initially successful impact of parasympathetic denervation. Hence, the discriminatory effect of persistently effective parasympathetic denervation might rather be seen later i.e. after 1 month [9] or three months post-ablation as performed in our study.

Patients should be well-informed about the likelihood of an increased heart rate post-PVI since a heart rate increase post-ablation was observed in over 80% of the patients in our study. However, while an increased heart rate post-ablation seems to be protective with respect to recurrences, it has also been connected to adverse cardiovascular outcomes in patients with but also without cardiovascular risk factors [15–18]. In this context, the extent but also the duration of an elevated heart rate might play an important role. Interestingly, in a study of Pappone et al*.* changes in heart rate returned to pre-ablation levels at six months [7]. In another study of Nilsson et al*.*, however*,* heart rate increases post-ablation persisted during the total follow-up period of 12 months (mean heart rate 70 bpm at 12 months compared to 58 bpm pre-ablation) [9]. Hence, those with persistently elevated heart rates and cardiovascular risk factors might benefit from a more intense beta-blocker therapy.

Treatment with beta-blocker as well as other AADs can modulate the sinus heart rate and, thus, are potential confounders for this study. However, the treatments with beta-blocker and other ADDs were similar in the analyzed patient groups without statistically significant differences (heart rate change < 11 bpm vs. >= 11 bpm and 1st quartile vs. 2nd–4th quartile at 3 M, respectively). Moreover, the results from the consistency analysis indicate that the observed association between heart rate change and late recurrences are independent of the use of beta-blocker or other AADs.

According to our data, a heart rate change < 11 bpm (PRE to 3 M) at 3 M is linked to an unfavorable outcome in terms of recurrences during one-year post-PVI. As an alternative parameter, patients with a heart rate < 60 bpm at 3 M are at higher risk for recurrences. This alternative marker might be relevant especially in settings where a PRE ECG is not available. Both parameters are clinically easily applicable tools which aid in identifying patients at high risk for recurrences benefitting from close monitoring. However, a combination with other ECG based markers is to be proven.

### Limitations

This is a registry-based cohort study. Moreover, as all data have been collected at a single electrophysiology center with local routines for ablation procedure, results may differ from other centres. Only about a fourth of the screened patients were included in the final analysis which could have led to a potential selection bias. All ablation procedures were performed applying radiofrequency energy and our results may not be applicable to other forms of energy delivery such as cryoablation or more complex ablation techniques. Patients were only selected when sinus rhythm was present at PRE, POST and 3 M. Therefore, we cannot firmly conclude on the ablation effect on heart rate in those with ongoing atrial fibrillation at PRE, POST and/or 3 M. Since follow-up post-ablation did not include intensive monitoring by transtelephonic monitoring or implantable loop recorder it is possible that asymptomatic recurrences may have been missed.

## Conclusion

Our study confirms the impact of PVI on cardiac autonomic function with a significant sinus heart rate increase post-ablation. Patients with a sinus heart rate change < 11 bpm (PRE to 3 M) are at higher risk for recurrences during one-year post-PVI. Hence, an extra-careful follow-up of these patients is advisable.

## Data availability statement

The data underlying this article cannot be shared publicly due to the privacy of individuals that were investigated in the study. The data will be shared on reasonable request to the corresponding author provided that this in accordance with the institutional ethical guidelines as well as regulation and legislation.

## Electronic supplementary material

Below is the link to the electronic supplementary material.

Supplementary file1 (PDF 22 KB) Figure 1 PRISMA flow diagram showing the selection process for all patients

Supplementary file2 (TIFF 13683 KB) Figure 2 Sinus heart rate change from PRE to POST (white bars) and PRE to 3M (grey bars)

Supplementary file3 (TIFF 13683 KB) Figure 3 Kaplan–Meier analysis of event-free survival from recurrence in patients within the 1st,2nd, 3rd and 4th quartile of sinus heart rate at 3M after a one-year follow-up

Supplementary file4 (TIFF 17718 KB) Figure 4 Mean sinus heart rate (with 95% confidence interval) of patients without any treatment of beta-blocker or AADs * p < 0.001 as compared to PRE, AAD = antiarrhythmic drug

Supplementary file5 (TIFF 17711 KB) Figure 5 Kaplan–Meier analysis of event-free survival from recurrence in patients not treated with any beta-blocker or AADs and a sinus heart rate change <11 bpm compared to >= 11 bpm (PRE to 3M) after a one-year follow-up

Supplementary file6 (DOCX 17 KB) Table 1 Logistic regression analysis for recurrence. BMI = body mass index, CI = confidence interval. Table 2 Logistic regression analysis for recurrence. BMI = body mass index, CI = confidence interval
