# Supplementary material for: Sinus heart rate post pulmonary vein ablation and long-term risk of recurrences
Source: Clin Res Cardiol. 2020 Nov 12;110(6):851–60. doi: 10.1007/s00392-020-01765-z (PMC8166690; doi:10.1007/s00392-020-01765-z)
Supplement: Supplementary file 1 — Supplementary file1 (PDF 22 KB) Figure 1 PRISMA flow diagram showing the selection process for all patients [file 392_2020_1765_MOESM1_ESM.pdf]

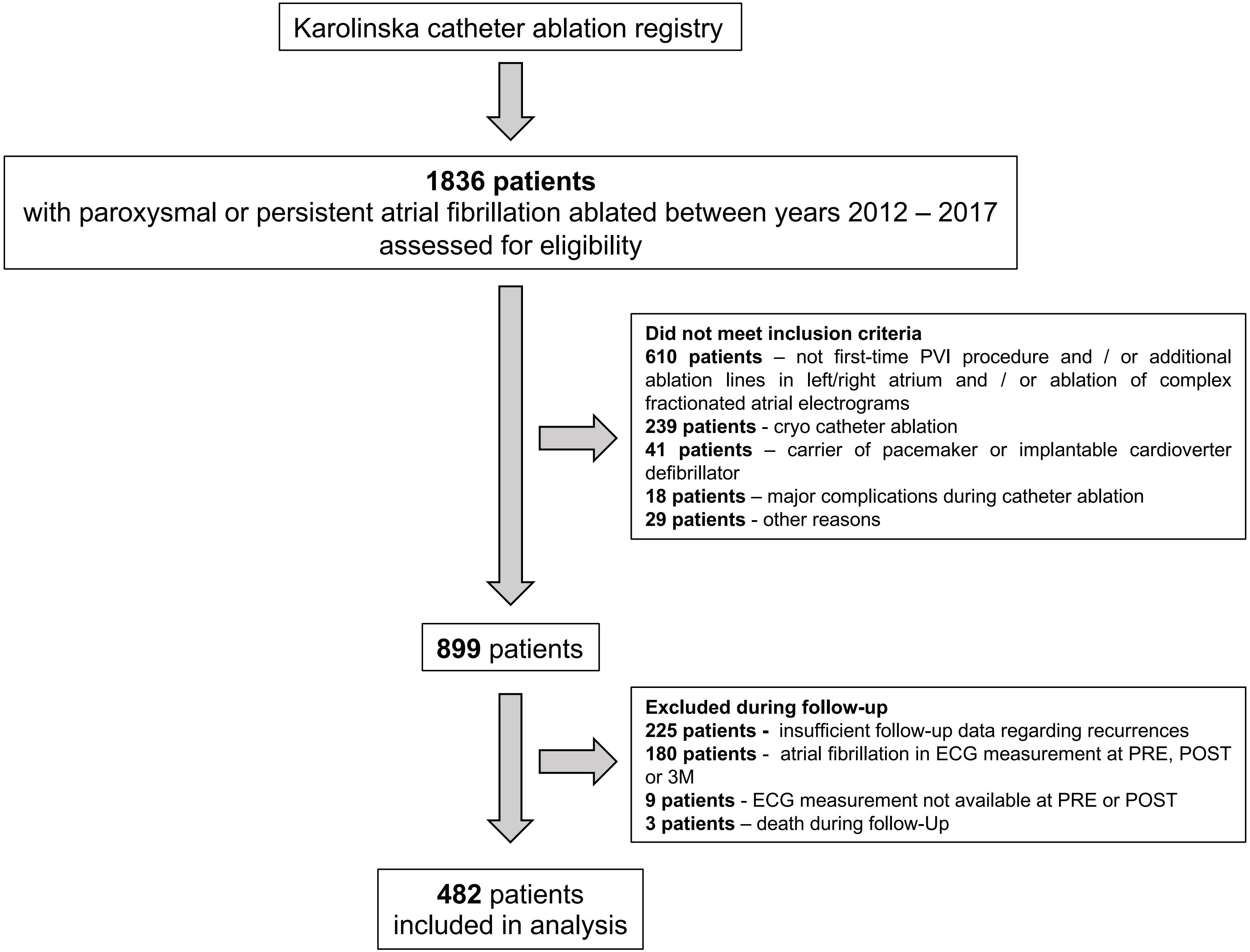

Karolinska catheter ablation registry
1836 patients
with paroxysmal or persistent atrial fibrillation ablated between years 2012 – 2017
assessed for eligibility
Did not meet inclusion criteria
610 patients – not first-time PVI procedure and / or additional ablation lines in left/right atrium and / or ablation of complex fractionated atrial electrograms
239 patients - cryo catheter ablation
41 patients – carrier of pacemaker or implantable cardioverter defibrillator
18 patients – major complications during catheter ablation
29 patients - other reasons
899 patients
Excluded during follow-up
225 patients - insufficient follow-up data regarding recurrences
180 patients - atrial fibrillation in ECG measurement at PRE, POST or 3M
9 patients - ECG measurement not available at PRE or POST
3 patients – death during follow-Up
482 patients
included in analysis
