# Supplementary figures and images for: Sinus heart rate post pulmonary vein ablation and long-term risk of recurrences
Source: Clin Res Cardiol. 2020 Nov 12;110(6):851–60. doi: 10.1007/s00392-020-01765-z (PMC8166690; doi:10.1007/s00392-020-01765-z)

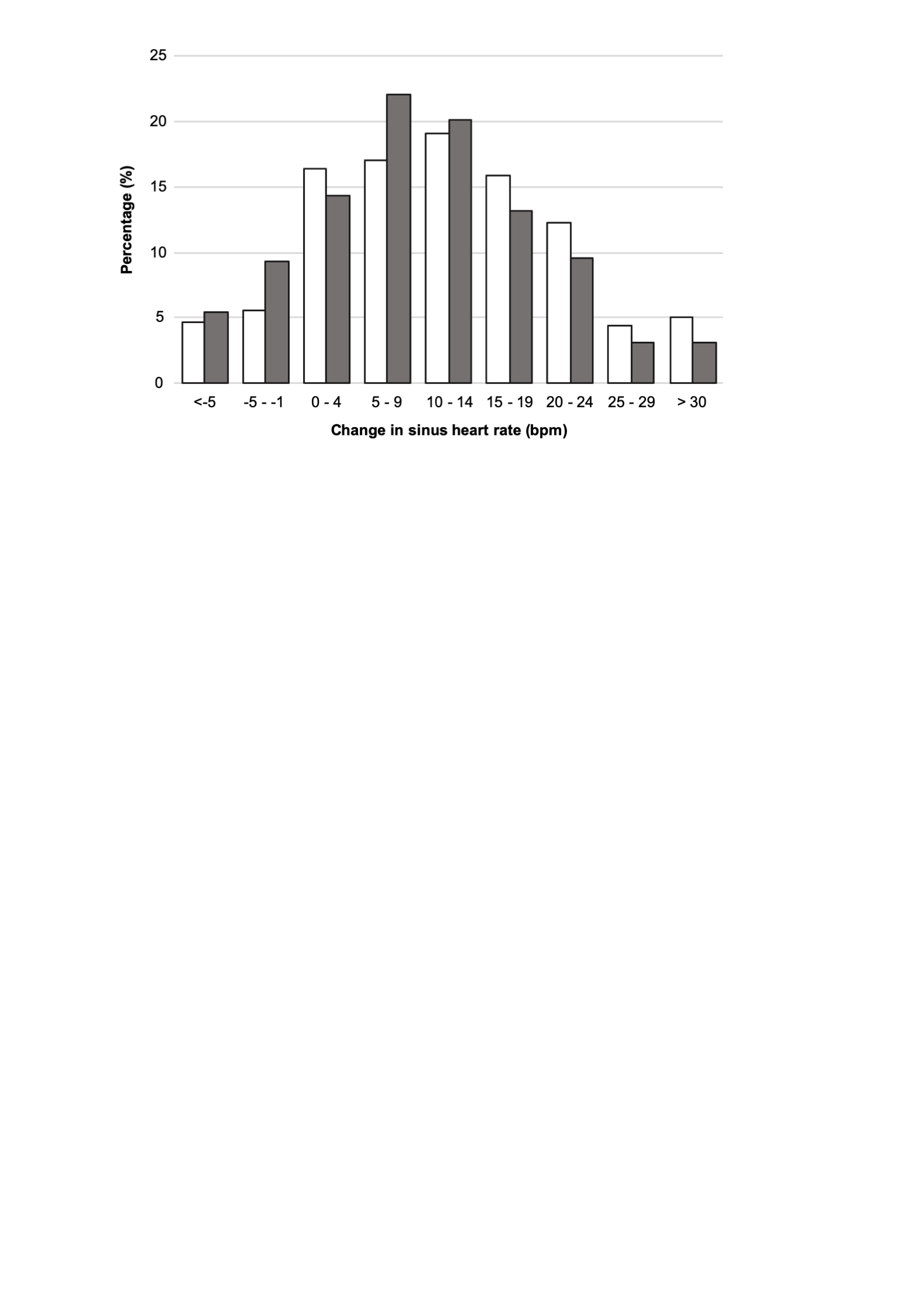

Supplement: Supplementary file 2 — Supplementary file2 (TIFF 13683 KB) Figure 2 Sinus heart rate change from PRE to POST (white bars) and PRE to 3M (grey bars) [file 392_2020_1765_MOESM2_ESM.tiff]

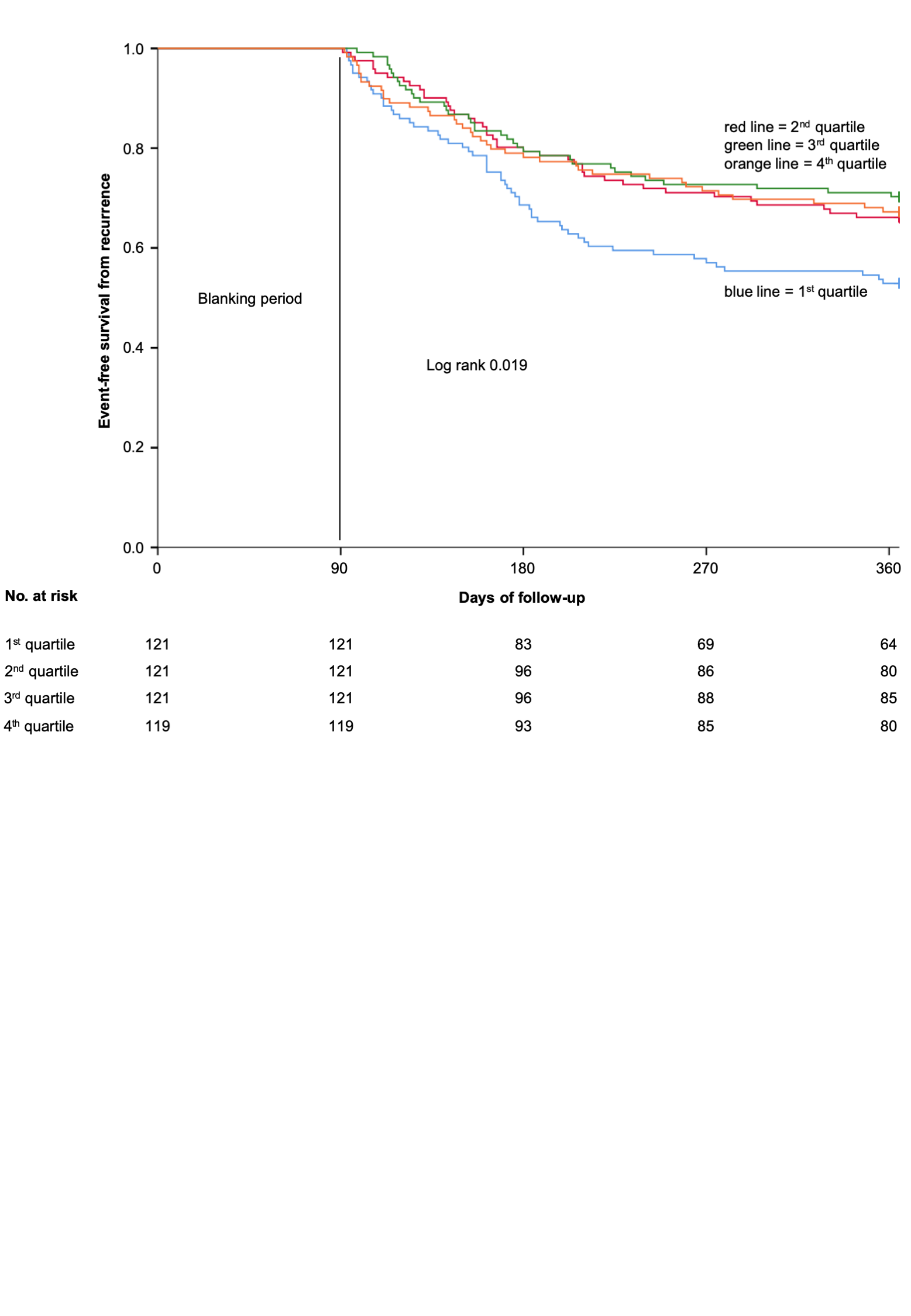

Supplement: Supplementary file 3 — Supplementary file3 (TIFF 13683 KB) Figure 3 Kaplan–Meier analysis of event-free survival from recurrence in patients within the 1st,2nd, 3rd and 4th quartile of sinus heart rate at 3M after a one-year follow-up [file 392_2020_1765_MOESM3_ESM.tiff]

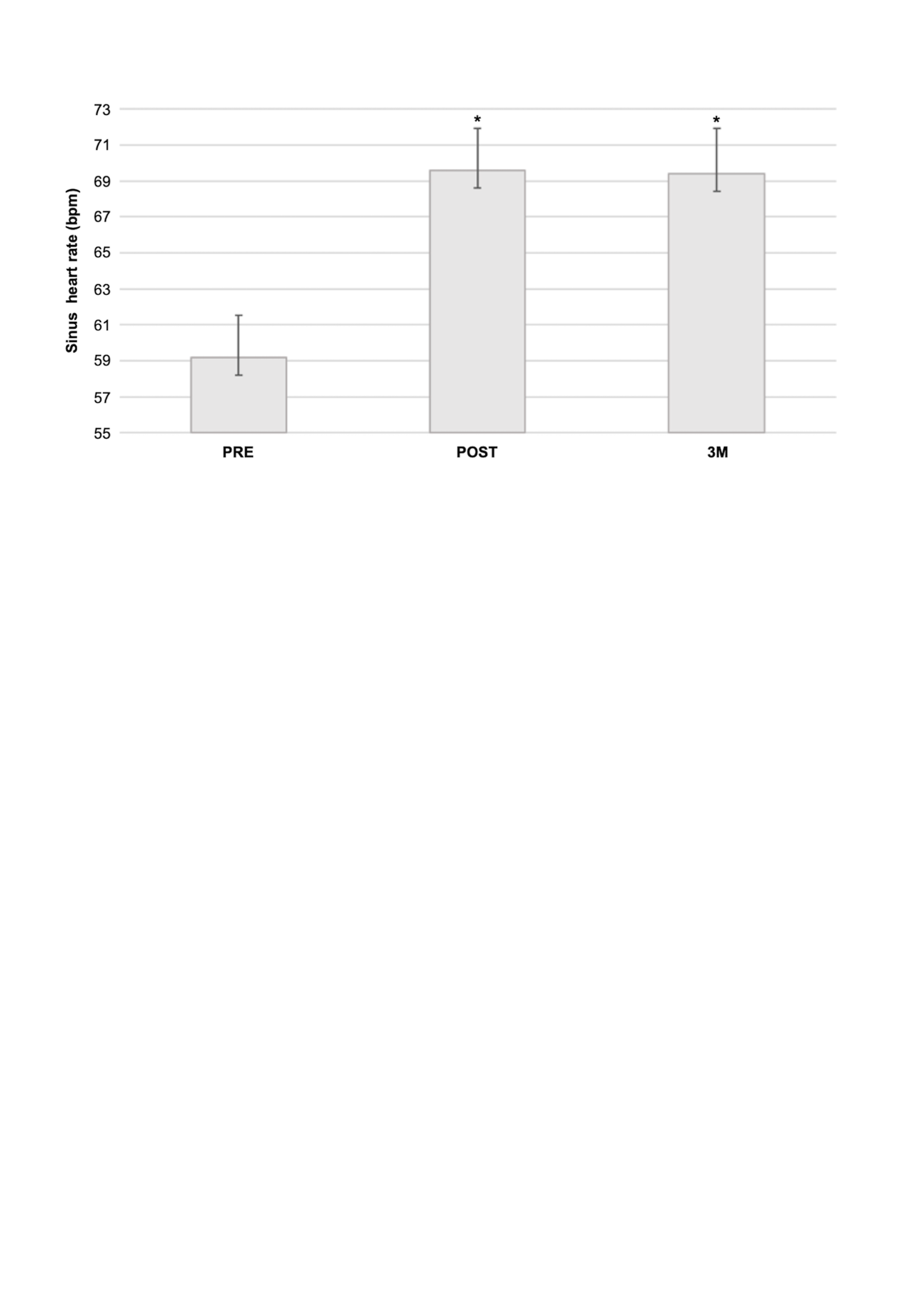

Supplement: Supplementary file 4 — Supplementary file4 (TIFF 17718 KB) Figure 4 Mean sinus heart rate (with 95% confidence interval) of patients without any treatment of beta-blocker or AADs * p < 0.001 as compared to PRE, AAD = antiarrhythmic drug [file 392_2020_1765_MOESM4_ESM.tiff]

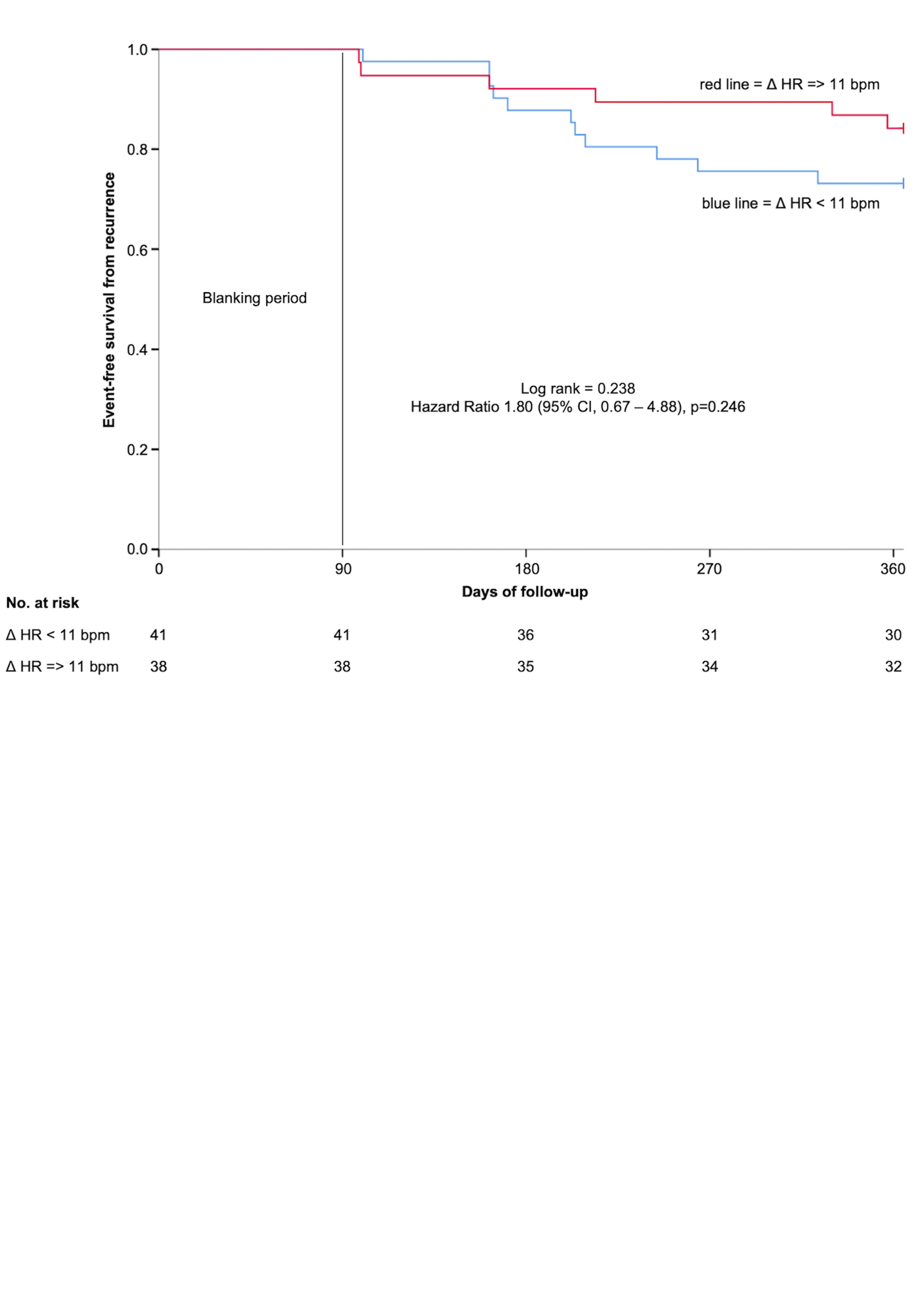

Supplement: Supplementary file 5 — Supplementary file5 (TIFF 17711 KB) Figure 5 Kaplan–Meier analysis of event-free survival from recurrence in patients not treated with any beta-blocker or AADs and a sinus heart rate change <11 bpm compared to >= 11 bpm (PRE to 3M) after a one-year follow-up [file 392_2020_1765_MOESM5_ESM.tiff]
