# Supplementary material for: Sinus heart rate post pulmonary vein ablation and long-term risk of recurrences
Source: Clin Res Cardiol. 2020 Nov 12;110(6):851–60. doi: 10.1007/s00392-020-01765-z (PMC8166690; doi:10.1007/s00392-020-01765-z)
Supplement: Supplementary file 6 — Supplementary file6 (DOCX 17 KB) Table 1 Logistic regression analysis for recurrence. BMI = body mass index, CI = confidence interval. Table 2 Logistic regression analysis for recurrence. BMI = body mass index, CI = confidence interval [file 392_2020_1765_MOESM6_ESM.docx]

Table 1, supplementary

Logistic regression analysis for recurrence

| **Univariate analysis** | | |
| --- | --- | --- |
| **Variable** | **Odds ratio (95% CI)** | **p-value** |
| BMI | 1.088 (1.031 – 1.148) | 0.002 |
| Persistent atrial fibrillation | 4.787 (3.118 – 7.351) | <0.001 |
| Duration of atrial fibrillation in the past | 1.009 (1.005 – 1.012) | <0.001 |
| Arterial hypertension | 1.789 (1.228 – 2.606) | 0.002 |
| Diabetes | 2.320 (1.088 – 4.947) | 0.029 |
| Left atrial size, parasternal long axis | 1.058 (1.012 – 1.106) | 0.013 |
| Procedure time | 1.005 (1.001 – 1.009) | 0.010 |
| beta-blocker at PRE | 1.497 (1.008 – 2.224) | 0.045 |
| beta-blocker at 3M | 1.787 (1.199 – 2.662) | 0.004 |
| other AADs at 3M |  |  |
| none | reference |  |
| Dronedarone, n (%) | 2.186 (0.999 - 4.785 | 0.050 |
| Flecainid, n (%) | 3.481 (1.339 – 9.046) | 0.010 |
| Heart rate change < 11 bpm (PRE to 3M) | 2.075 (1.410 – 3.054) | <0.001 |
| **Multivariable analysis** | | |
| Persistent atrial fibrillation | 4.152 (2.638 – 6.534) | <0.001 |
| Diabetes mellitus | 2.875 (1.237 – 6.680) | 0.014 |
| Heart rate change < 11 bpm (PRE to 3M) | 1.747 (1.140 – 2.677) | 0.010 |
| beta-blocker at 3M | 1.714 (1.096 – 2.680) | 0.018 |
| Duration of atrial fibrillation in the past | 1.008 (1.004 – 1.011) | <0.001 |
| Procedure time | 1.004 (1.000 – 1.008) | 0.046 |

BMI = body mass index, CI = confidence interval

Table 2, supplementary

Logistic regression analysis for recurrence

| **Univariate analysis** | | |
| --- | --- | --- |
| **Variable** | **Odds ratio (95% CI)** | **p-value** |
| BMI | 1.088 (1.031 – 1.148) | 0.002 |
| Persistent atrial fibrillation | 4.787 (3.118 – 7.351) | <0.001 |
| Duration of atrial fibrillation in the past | 1.009 (1.005 – 1.012) | <0.001 |
| Arterial hypertension | 1.789 (1.228 – 2.606) | 0.002 |
| Diabetes | 2.320 (1.088 – 4.947) | 0.029 |
| Left atrial size, parasternal long axis | 1.058 (1.012 – 1.106) | 0.013 |
| Procedure time | 1.005 (1.001 – 1.009) | 0.010 |
| beta-blocker at PRE | 1.497 (1.008 – 2.224) | 0.045 |
| beta-blocker at 3M | 1.787 (1.199 – 2.662) | 0.004 |
| other AADs at 3M |  |  |
| none | reference |  |
| Dronedarone, n (%) | 2.186 (0.999 - 4.785 | 0.050 |
| Flecainid, n (%) | 3.481 (1.339 – 9.046) | 0.010 |
| Heart rate < 60 bpm at 3M | 1.881 (1.236 – 2.862) | 0.003 |
| **Multivariable analysis** | | |
| Persistent atrial fibrillation | 4.317 (2.740 – 6.801) | <0.001 |
| Diabetes mellitus | 2.935 (1.273 – 6.764)) | 0.011 |
| Heart rate < 60 bpm at 3M | 1.954 (1.229 – 3.105) | 0.005 |
| beta-blocker at 3M | 1.740 (1.112 – 2.722) | 0.015 |
| Duration of atrial fibrillation in the past | 1.008 (1.004 – 1.012) | <0.001 |
| Procedure time | 1.004 (1.000 – 1.008) | 0.039 |

BMI = body mass index, CI = confidence interval
